# Computer face-matching technology using two-dimensional photographs accurately matches the facial gestalt of unrelated individuals with the same syndromic form of intellectual disability

**DOI:** 10.1186/s12896-017-0410-1

**Published:** 2017-12-19

**Authors:** Tracy Dudding-Byth, Anne Baxter, Elizabeth G. Holliday, Anna Hackett, Sheridan O’Donnell, Susan M. White, John Attia, Han Brunner, Bert de Vries, David Koolen, Tjitske Kleefstra, Seshika Ratwatte, Carlos Riveros, Steve Brain, Brian C. Lovell

**Affiliations:** 1 Hunter Genetics, Hunter New England Health Service, Newcastle, NSW Australia; 2 GrowUpWell, Priority of Research Excellence, The University of Newcastle, Newcastle, NSW Australia; 3 Hunter Medical Research Institute, Newcastle, NSW Australia; 4 The University of Newcastle, Newcastle, NSW Australia; 5 Murdoch Children’s Research Institute, Melbourne, Victoria Australia; 6 Department of Paediatrics, University of Melbourne, Melbourne, Victoria Australia; 7 Department of Human Genetics and Donders Institute for Brain, Cognition and Behaviour, Radboud University Medical Center, Nijmegen, the Netherlands; 8 The Department of Medicine, John Hunter Hospital, Newcastle, NSW Australia; 9 Imagus Technology, Brisbane, QLD Australia; 10 School of ITEE, The University of Queensland, Brisbane, QLD Australia; 11 New South Wales Genetics of Learning Disability (GOLD) service, Hunter New England Health Service, Newcastle, NSW 2298 Australia

**Keywords:** 2D photography, Clinical genetics, Computer vision, Computational biology, Dysmorphology, Facial gestalt, Intellectual disability, Syndromic, Phenotyping

## Abstract

**Background:**

Massively parallel genetic sequencing allows rapid testing of known intellectual disability (ID) genes. However, the discovery of novel syndromic ID genes requires molecular confirmation in at least a second or a cluster of individuals with an overlapping phenotype or similar facial gestalt. Using computer face-matching technology we report an automated approach to matching the faces of non-identical individuals with the same genetic syndrome within a database of 3681 images [1600 images of one of 10 genetic syndrome subgroups together with 2081 control images]. Using the leave-one-out method, two research questions were specified:

1. Using two-dimensional (2D) photographs of individuals with one of 10 genetic syndromes within a database of images, did the technology correctly identify more than expected by chance: i) a top match? ii) at least one match within the top five matches? or iii) at least one in the top 10 with an individual from the same syndrome subgroup?
2. Was there concordance between correct technology-based matches and whether two out of three clinical geneticists would have considered the diagnosis based on the image alone?

**Results:**

The computer face-matching technology correctly identifies a top match, at least one correct match in the top five and at least one in the top 10 more than expected by chance (*P* < 0.00001). There was low agreement between the technology and clinicians, with higher accuracy of the technology when results were discordant (*P* < 0.01) for all syndromes except Kabuki syndrome.

**Conclusions:**

Although the accuracy of the computer face-matching technology was tested on images of individuals with known syndromic forms of intellectual disability, the results of this pilot study illustrate the potential utility of face-matching technology within deep phenotyping platforms to facilitate the interpretation of DNA sequencing data for individuals who remain undiagnosed despite testing the known developmental disorder genes.

**Electronic supplementary material:**

The online version of this article (10.1186/s12896-017-0410-1) contains supplementary material, which is available to authorized users.

## Background

Intellectual disability (ID) poses a significant psychological and economic burden on families with 1.5–2% of all children having an intellectual quotient (IQ) < 70 and 0.3–0.5% of children having moderate-severe ID with an IQ < 50 [1, 2]. Although a pharmacological therapy is only available for the minority of individuals with rare metabolic diseases, a definitive diagnosis can inform prognosis, guide management and restore reproductive confidence for parents planning further children. Understanding the genetic basis of ID is the first step towards understanding interacting biological pathways and possible targeted therapy.

A craniofacial anomaly is described in 30–50% of the known genetic causes of ID [3], and the specialty of clinical dysmorphology evolved as clinicians realised that some individuals with ID had a recognisable gestalt. Historically, the process of syndrome characterisation commenced with the publication of one or two individuals with a unique pattern of features. This facilitated the identification of other individuals with a similar constellation of features or characteristic gestalt, and over time a recognisable syndrome phenotype emerged [4]. The discovery of the molecular or biochemical basis for a particular condition, such as velocardiofacial syndrome and Smith-Lemli-Optiz syndrome, allowed characterization of the wider phenotypic spectrum. The overlapping facial gestalt of neurofibromatosis type 1, Costello, Noonan, cardiofaciocutaneous and LEOPARD syndromes led to the identification of genes within a common developmental RASopathy pathway [5].

A clinical dysmorphologist is trained to recognise the typical gestalt of a well described condition, but a clinical diagnosis is more difficult when clinical features are outside the recognised spectrum or the characteristic phenotype is altered by ethnicity or age. For many rare conditions, a clinical dysmorphologist may have never seen another child with the same condition.

Recent advances in high-throughput genetic sequencing now allows rapid testing of all the known ID genes in a single test. However, the question remains on how best to proceed when a child with syndromic ID remains undiagnosed despite testing all the known developmental disorder genes. The comparison of exome sequence data from as few as two unrelated individuals with the same clinical phenotype has revolutionised novel ID gene discovery. However, characterising a novel syndromic form of ID still requires clinicians to initially locate at minimum a second individual or a cluster of individuals with a similar facial gestalt through a process of presenting photographs at clinical dysmorphology meetings internationally; or using databases linked to Matchmaker Exchange [6] to identify individuals with variants in the same candidate gene.

A number of researchers have explored the option of automated facial analysis using three-dimensional (3D) digital imaging and 2D computer systems for dysmorphology and facial phenotyping [7–15]. While 3D imaging overcomes variations in pose, distance and illumination, it requires specialised 3D capturing equipment and is not currently practical in the clinic setting. Boehringer et al. subjected 2D photographs to graph based analysis [10, 13] with 21% classification accuracy in the clinic setting [11]. Ferry et al. developed a computer based model to identify the patterns of facial abnormalities on 2D photographs [15]. The method published by Ferry et al. uses active appearance models (AAM) to label key-points on the face which performs well on high quality images.

The face recognition algorithm used in this project was initially developed to match the facial images of individuals for the primary purpose of recognising blurry faces in CCTV for policing and counterterrorism. The algorithm was trained on over 3 million faces and is based on the latest state-of–the-art deep learning techniques. Benchmark algorithm performance for facial recognition is based on the Face Recognition Grand Challenge (FRGC) dataset with 16,028 face images from 4007 subjects. FRGC was proposed by the National Institute of Standards and Technology (NIST) to promote and advance face recognition technology designed to support face recognition efforts in the U.S. Government [16]. This dataset contains images acquired from both controlled and uncontrolled environments. A standard way to compare biometric systems is to measure their False Reject Rate (FRR) at the standard False Acceptance Rate (FAR) of 0.001 or 0.1%. With CCTV or multiple image enrolment, the error rate of our algorithm on the FRGC benchmark faces can be considered as negligible (0.01%). (Additional file 1).

Commercial versions of this software are deployed at sites such as the Swinburne University of Technology, Melbourne where it is currently used to detect persons of interest from CCTV streams. The software is also being used to detect persons of interest in large crowd gatherings through various police and other security agencies. Since the software was developed for CCTV, high quality professional photographs are not required and even poor quality historical photographs can readily be used. This technology is unique as it uses low-resolution structural and frequency domain features rather than high resolution features. It is based on spatial textures and statistical models and is simultaneously insensitive to pose, illumination, expression, obscuration, blurring, decoding artefacts, and low-resolution images [17, 18]. Testing on the Labelled Faces in the Wild dataset of celebrity photographs showed strong matching between relatives, which led to the hypothesis that high rank matches could be strongly indicative of close DNA matching [19].

The aim of this pilot study was to explore the accuracy of this robust computer face-matching technology (FMT) for matching the faces of non-identical individuals with the same genetic syndrome diagnosis.

## Methods

Accuracy of the FMT was based on the software’s ability to match facial images of unrelated individuals from each of 10 different syndrome subgroups to a reference bank of images; this reference bank contained 1600 images of children and adults with one of the 10 syndromic forms of ID [653 from manuscripts, 698 from the internet and 249 patients with Cornelia de Lange syndrome provided from a cohort published by Ferry et al. 2014 together with 2081 controls [20] (Table 1).

**Table 1:** Number of images in the database at different stages of analysis

	Syndrome	Images added to database before this stage of analysis	Number of images for syndrome	Total images in database at time of analysis
1	Williams	1	183	3145
1	Rubinstein-Taybi	1	155	3145
1	Floating Harbor	1	61	3145
1	Coffin Lowry	1	154	3145
1	Kabuki	1	195	3145
1	Smith Magenis	1	124	3145
2	PACS1	2	39	3432
2	Kleefstra	2	128	3432
2	Koolen-de Vries	2	120	3432
3	Cornelia de Lange	192 before Stage 1, then an additional 249 before Stage 3	441	3681

A PubMed search was performed for each syndrome, and images were downloaded from all peer reviewed publications containing facial images. Images from the internet were curated by clinical geneticists [TD and AH]. Tinyurl links to sources for the database are available at Open Science Framework (OSF) as FaceDx project. Links are expected to decay with time. Applications for the full dataset can be made to the corresponding author.

Images were captured as JPEG files, and labelled with syndrome diagnosis and image number prior to being uploaded into the database. Multiple images of the same individual at the same age were not collected; however, there were occasions where multiple images of the same individual at different ages were included and numbered accordingly (for example Coffin-Lowry patient 1.1, 1.2 and 1.3). No restrictions were placed on variations in photograph quality, pose, face rotation, lighting, facial expression, individual age, gender or ethnicity. The stages of analysis are shown in Table 1. At Stage 1, the database contained images for all controls and 6 syndromes: Williams, Rubinstein-Taybi, Floating Harbor, Coffin-Lowry, Kabuki and Smith Magenis. Each syndrome was analysed individually. At this stage, the database also contained 192 images for Cornelia de Lange syndrome; however, analysis of this syndrome was delayed until Stage 3 (see below). After Stage 1 analyses, images for an additional three syndromes were added to the database and Stage 2 analyses were performed. Finally, images for an additional 249 patients with Cornelia de Lange syndrome provided from a cohort published by Ferry et al. [15] were added to the database (which already contained images for 192 Cornelia de Lange patients) and analyses of this syndrome was performed (Stage 3). The accuracy of the FMT was tested using the leave-one-out method, i.e., removing an individual image from the database and letting the software list the top 10 closest matches when the removed image is used as the test case. We recorded whether another non-identical individual with the same syndrome diagnosis was the closest match, within the top five closest matches or within the top 10 closest matches. In the situation where there were multiple photographs of the same individual at different ages, all photographs were removed from the database, to ensure that the test case didn’t match with an image of them at a different age. Our secondary analysis aimed to compare the accuracy of the software diagnosis with that of a clinical geneticist. Three clinical geneticists were given the diagnosis for each of the 10 syndromes and asked to score the likelihood that they would have made that particular syndrome diagnosis based on the photograph alone (1 = definitely would have considered this diagnosis based on the photograph alone; 2 = unlikely to have considered this diagnosis based on the photograph alone and 3 = possibly would have considered this diagnosis based on the photograph alone). As there were only 10 syndromes, the clinical geneticists could not be blinded to the actual diagnosis, but they were blinded to the results of the FMT and the scores of each other. For this pilot study, the clinicians were not asked to score the control images as non-syndromic. Advice from the Hunter New England Health Research Ethics Committee concluded that the use of publicly available images did not require special consent.

### Statistical analysis

Two research questions were analysed separately for each syndrome. Firstly, for each of the syndrome-specific individuals, using all other facial images present in the database, did the software correctly identify a match from the same syndrome subgroup more often than expected by chance? Three definitions of a “match” were used: i) same syndrome as top match; ii) same syndrome within the top five matches; and iii) same syndrome within the top 10 matches. For each outcome, observed and expected frequencies of matches were compared by calculating a Chi-Square Goodness-of-t statistic, applying Yates’ continuity correction. Expected counts were estimated via simulation. Secondly, for each of the syndrome-specific individuals, what was the concordance between correct software-based matches (using each of the three definitions of a match) and clinical based-diagnosis? This analysis was performed in two parts, corresponding to two alternate definitions of a clinician-based diagnosis: 1) Whether at least two of the three clinicians said they would have considered a diagnosis of the syndrome based on the photograph alone; and 2) Whether all three clinicians would have considered a diagnosis of the syndrome based on the photograph alone.

For each of the two alternate clinical definitions, the three software-based outcomes (top 1, top 5 and top 10) were individually assessed (corresponding to six distinct analyses). For each of the six combinations of clinician and software-based diagnoses, 2 × 2 contingency tables were constructed showing frequencies for the paired outcomes (diagnostic ratings). Table frequencies were assessed for equality of row and column marginal frequencies using McNemar’s test and its associated *p*-value. A Kappa statistic for agreement was also calculated. For all analyses, results with *p* < 0.05 were considered significant. All statistical analyses were programmed using SAS v9.4 (SAS Institute, Cary, North Carolina, USA).

## Results

### Analysis 1: Comparing observed to expected frequencies of correct software matches.

Tables 2, 3 and 4 show observed and expected frequencies of syndromic patients for whom the top match, at least one in the top five matches, and at least one in the top 10 matches, were from the relevant syndrome subgroup, respectively. In each case, observed and expected frequencies were compared by calculating a Chi-square statistic and its associated p-value. For all syndromes, and using all three definitions of a match, the software matched syndromic patients significantly more often than expected by chance. Chi-square statistics were > 800, > 350 and > 150, respectively, with *P* values < 0.00001.

**Table 2:** Observed and expected counts of individuals with a syndrome diagnosis for whom the top match was another unrelated individual within the same syndrome subgroup

Syndrome	Number of images for syndrome	Observed match/No match	Expected match/No match	Chi-square (1)	*P*-value
Williams	183	110/73	11/172	938.43	<.00001
Rubinstein-Taybi	155	92/63	7/148	1068.28	<.00001
Floating Harbor	61	42/19	2/59	806.57	<.00001
Coffin Lowry	154	88/66	7/147	969.83	<.00001
Kabuki	195	106/89	12/183	776.29	<.00001
Smith Magenis	124	72/52	5/119	921.61	<.00001
PACS1	39	6/33	2/37	6.46	0.01106
Kleefstra	128	72/56	5/123	920.40	<.00001
Koolan-de Vries	120	66/54	4/116	978.17	<.00001
Cornelia de Lange	441	329/112	53/388	1627.70	<.00001

**Table 3:** Observed and expected counts of individuals with a syndrome diagnosis for whom at least one in the top five matches was another unrelated individual within the same syndrome subgroup

Syndrome	Number of images for syndrome	Observed match/No match	Expected match/No match	Chi-square (1)	*P*-value
Williams	183	176/7	47/136	472.74	<.00001
Rubinstein-Taybi	155	149/6	34/121	493.94	<.00001
Floating Harbor	61	58/3	5/56	600.47	<.00001
Coffin Lowry	154	133/21	34/120	366.21	<.00001
Kabuki	195	187/8	53/142	461.78	<.00001
Smith Magenis	124	122/2	23/101	517.90	<.00001
PACS1	39	19/20	3/36	86.76	<.00001
Kleefstra	128	108/20	22/106	401.25	<.00001
Koolen-de Vries	120	96/24	20/100	342.02	<.00001
Cornelia de Lange	441	418/23	208/233	399.38	<.00001

**Table 4:** Observed and expected counts of individuals with a syndrome diagnosis for whom at least one in the top ten matches was another unrelated individual within the same syndrome subgroup

Syndrome	Number of images for syndrome	Observed match/No match	Expected match/No match	Chi-square (1)	*P*-value
Williams	183	179/4	82/101	205.76	<.00001
Rubinstein-Taybi	155	155/0	62/93	230.01	<.00001
Floating Harbor	61	58/3	11/50	239.81	<.00001
Coffin Lowry	154	146/8	61/93	193.83	<.00001
Kabuki	195	192/3	92/103	203.73	<.00001
Smith Magenis	124	123/1	41/83	242.03	<.00001
PACS1	39	28/11	4/35	153.84	<.00001
Kleefstra	128	119/9	40/88	224.08	<.00001
Koolan-de Vries	120	109/11	36/84	208.58	<.00001
Cornelia de Lange	441	432/9	317/124	147.09	<.00001

### Analysis 2: Comparing the accuracy of software-based and clinician diagnoses

Table 5 shows frequencies from the 2 × 2 contingency tables and results of McNemar’s test comparing concordance between two alternate diagnostic ratings for each patient: whether the software identified a top match from the same syndrome and if at least two of three clinicians would have considered a diagnosis of the syndrome based on the photograph alone. Also shown are kappa statistics representing the agreement between software and clinician diagnoses. Kappa statistics were low, reflecting poor agreement between software and clinician diagnoses. Frequencies of the two discordant cells were significantly different for five of the 10 disorders. For four of these (Coffin-Lowry, Smith Magenis, PACS1 and Kleefsta syndrome), the software correctly classified the syndrome more often than the clinicians did. The striking exception was Kabuki syndrome, for which the clinicians performed markedly better than the software.

**Table 5:** Concordance between a software-identified top match and whether two of three clinical geneticists would definitely have considered this diagnosis based on the image alone

Syndrome	Frequency	Correctly classified by neither	Correctly classified by both	Correctly classified by software, but not clinicians	Correctly classified by clinicians, but not software	McNemars Chi-square	*p*-value	Kappa
Williams	183	31	66	44	42	0.05	0.82925	0.02
Rubinstein-Taybi	155	28	61	31	35	0.24	0.62246	0.11
Floating Harbor	61	7	33	9	12	0.43	0.51269	0.16
Coffin Lowry	154	42	41	47	24	7.45	0.00634	0.10
Kabuki	195	23	88	18	66	27.43	<.00001	0.09
Smith Magenis	124	34	38	34	18	4.92	0.02650	0.17
PACS1	39	33	2	4	0	4.00	0.04550	0.46
Kleefstra	128	52	9	63	4	51.96	<.00001	0.05
Koolan-de Vries	120	30	37	29	24	0.47	0.49221	0.12
Cornelia de Lange	441	52	264	65	60	0.20	0.65472	0.26

Table 6 shows contingency table frequencies and results of McNemar’s test comparing concordance between two alternate diagnostic ratings for each patient: whether the software identified at least one patient with the same syndrome within the top five matches and at least two clinicians would have considered a diagnosis of the syndrome. For statistical analysis, the software was recorded as “making a diagnosis” if there was an individual within the same syndrome subgroup within the top 5 closest matches within a dataset of 3681 images. Kappa statistics were low, reflecting poor agreement between software and clinician diagnoses. Frequencies of the two discordant cells were significantly different for all 10 disorders. In all cases, the software correctly classified the syndrome more often than the clinicians did.

**Table 6:** Concordance between a software-identified match within the top five closest matches and whether two of three clinical geneticists would definitely have considered this diagnosis based on the image alone

Syndrome	Frequency	Correctly classified by neither	Correctly classified by both	Correctly classified by software, but not clinicians	Correctly classified by clinicians, but not software	McNemars Chi-square	*p*-value	Kappa
Williams	183	2	103	73	5	59.28	<.00001	−0.02
Rubinstein-Taybi	155	4	94	55	2	49.28	<.00001	0.06
Floating Harbor	61	1	43	15	2	9.94	0.00162	0.02
Coffin Lowry	154	15	59	74	6	57.80	<.00001	0.07
Kabuki	195	2	148	39	6	24.20	<.00001	0.01
Smith Magenis	124	2	56	66	0	66.00	<.00001	0.03
PACS1	39	20	2	17	0	17.00	0.00004	0.11
Kleefstra	128	18	11	97	2	91.16	<.00001	0.00
Koolan-de Vries	120	17	54	42	7	25.00	<.00001	0.18
Cornelia de Lange	441	10	311	107	13	73.63	<.00001	0.06

Table 7 shows contingency table frequencies and results of McNemar’s test comparing concordance between two alternate diagnostic ratings for each patient: whether the software identified at least one patient with the same syndrome within the top 10 matches and at least two clinicians would have considered a diagnosis of the syndrome. Kappa statistics were low, reflecting poor agreement between software and clinician diagnoses. Frequencies of the two discordant cells were significantly different for nine of the 10 disorders. For RTS, McNemar’s chi-square statistic could not be calculated due to 0 cell counts in the denominator. In all cases, the software again correctly classified the syndrome more often than the clinicians did.

**Table 7:** Concordance between a software-identified match within the top ten closest matches and whether two of three clinical geneticists would definitely have considered this diagnosis based on the image alone

Syndrome	Frequency	Correctly classified by neither	Correctly classified by both	Correctly classified by software, but not clinicians	Correctly classified by clinicians, but not software	McNemars Chi-square	*p*-value	Kappa
Williams	183	1	105	74	3	65.47	<.00001	−0.02
Rubinstein-Taybi	155	0	96	59	0	.	.	0.00
Floating Harbor	61	1	43	15	2	9.94	0.00162	0.02
Coffin Lowry	153	5	63	83	2	77.19	<.00001	0.02
Kabuki	195	1	152	40	2	34.38	<.00001	0.02
Smith Magenis	124	1	56	67	0	67.00	<.00001	0.01
PACS1	39	11	2	26	0	26.00	<.00001	0.04
Kleefstra	128	9	13	106	0	106.00	<.00001	0.02
Koolan-de Vries	120	8	58	51	3	42.67	<.00001	0.09
Cornelia de Lange	441	6	321	111	3	102.32	<.00001	0.06

The analysis was repeated comparing the accuracy of the software-based and clinician diagnosis where all three clinical geneticists would have considered the relevant diagnosis based on the photograph alone. Additional file 2: Tables S8, S9 and S10 are included in the supplementary data. Kappa statistics were low, reflecting poor agreement between software and clinician diagnoses. Frequencies of the two discordant cells were significantly different for all 10 disorders. As was the case in Tables 6 and 7, the software correctly classified the syndrome more often than the clinicians did.

## Discussion

Prior to the availability of high-throughput massively parallel sequencing (MPS), the diagnostic rate for children with ID and dysmorphic facial features was less than 20% [21, 22]. The introduction of ID gene panels and exome sequencing, whereby known ID genes within an individual’s exome can be rapidly and simultaneously sequenced, has revolutionised clinical practice and increased the molecular diagnostic rate by 25–30% [23–25]. Whole-genome sequencing (WGS), which provides a complete view of the human genome, has a reported diagnostic rate for children with ID in one study of 42% [26]. However, when a pathogenic variant is not identified within the known developmental disorder genes [27], the enormous amount of sequence data generated by whole exome sequencing (WES) or WGS poses considerable challenges for analysis and interpretation. Standard pipelines with sophisticated filtering processes using public variant databases can reduce the ~20,000 coding variants identified by WES to <500 rare variants (≤ 1% in controls) per exome [24]. WES generates ~ 3 million variants compared to the reference genome. Considering that an estimated 75% of known rare diseases have a prevalence of 0.1–10 per 100,000 [28] locating a second patient with a similar phenotype is a rate-limiting step in ID gene discovery.

The need for detailed phenotyping in the era of MPS has led to deep phenotyping projects based on the human phenotype ontology [29] and international collaborative initiatives such as Decipher [30], phenomecentral [31], Genematcher [32], mygene2 [33] and Matchmaker Exchange [6]. In addition to a range of physical, cognitive and behavioural characteristics, deep phenotyping software requires the clinician to accurately observe, interpret and record facial morphology data, the reproducibility of which is subjective and can be limited by human variation and error.

The FMT used in this study is well suited to the clinical genetic setting, as it already performs live video recognition from mobile devices. Computer FMT can rapidly compare the test case against thousands of facial images within the database; whereas, a human is unable to remember all the faces when required to compare a facial image against a large number of images. The scalability of this technology on an international basis has the potential to enhance the efficacy of deep phenotyping platforms, used for the interpretation of sequencing data, by combining facial images with human phenome ontology terms. We acknowledge that a facebase will need to be populated with a large number of undiagnosed patients before a high-probability face-match can be made. For this reason, the next phase of our research will include clustering facial images, combined with human phenome ontology terms, within groups of genetically heterogeneous syndromic forms of ID. This will include a group of individuals with a clinical syndrome diagnosis who have tested negative for pathogenic variants within the genes currently known to cause the syndrome phenotype.

One limitation of this study is that we did not do a direct comparison between software and clinician accuracy at making a diagnosis and relied on retrospective diagnostic scoring of images by clinical geneticists [TD, AH, SW] or [BD, DK, TK]. Although the clinicians were aware of the diagnosis when scoring the likelihood of making a diagnosis, we attempted to minimise this bias by having three clinical geneticists independently provide a diagnosis likelihood score. The fact that the clinicians performed markedly better than the software for Kabuki syndrome is not surprising as the characteristic facial gestalt of Kabuki syndrome (resemblance to stage makeup used in traditional Japanese theatre called Kabuki) is very familiar to clinicians trained in clinical dysmorphology. Although the FMT performed better than clinicians for Coffin-Lowry and Smith Magenis syndrome based on the face alone, these diagnoses are often informed clinically by family history and behavioural phenotype. The majority of clinicians would have limited experience diagnosing the recently characterised Kleefstra syndrome and PACS1. All the images from published journals and the internet were curated by two authors (TD & AH). However, a confirmatory molecular diagnosis was unavailable for the majority of images within the database, and it is probable that the underlying molecular mechanism is variable in some individuals with Cornelia de Lange, Kleefstra or Koolen-de Vries syndromes. We did not make use of the number of times a syndrome was within the top 10 matches. For this current study, all images were loaded into the matching software manually one by one; however, we do plan to address this question using the automated version 2 of the software. This new version will allow images to be rapidly uploaded, generating a matching score and rank list of closest matches against every existing image in the database. Automation will also facilitate a large-scale project directly comparing the accuracy of the software with that of a group of clinical geneticists. We acknowledge the limited dataset used in this pilot study and the possibility that the accuracy of the face-matching technology may be influenced by the number of images within the facebase. Linking facial images of individuals within the database with their phenotypic and genetic data will allow filtered searches based on human phenome ontology terms and/or genetic testing. We have been unable to do a direct comparison with the facial dysmorphology novel analysis (FDNA) technology used by Face2Gene [34] as the images within our cohort are very likely to have been used to develop the FDNA diagnostic heat maps. Rather than developing a diagnostic tool based on a canonical face, our software generates a matching score against all other images within the database. This technology, therefore, complements the FDNA technology by providing face-matching across a broad spectrum of age, severity and ethnicity.

## Conclusions

Although the accuracy of the computer FMT was tested on images of individuals with known syndromic forms of ID, the results of this pilot study warrant further research into the utility of face-matching technology combined with deep phenotyping (based on human phenome ontology terms) in the interpretation of DNA sequencing data for individuals who remain undiagnosed despite testing the known developmental disorder genes.

## Additional files

Additional file 1: Imagus FRT Algorithm Performancev1.1.BL.pdf. Data on Face Recognition Algorithm Performance on Face Recognition Grand Challenge (FRGC) Benchmark. A license agreement to use FRGC database was obtained. (DOCX 139 kb)

Additional file 2: Table S8. Concordance between a software-identified top match and whether all three clinical geneticists would have considered this diagnosis based on the image alone. **Table S9.** Concordance between a software-identified match within the top five closest matches and whether all three clinical geneticists would have considered this diagnosis based on the image alone. **Table S10.** Concordance between a software-identified match within the top five closest matches and whether all three clinical geneticists would have considered this diagnosis based on the image alone. (DOCX 17 kb)

## Abbreviations

2D: Two-dimensional
3D: Three-dimensional
FMT: Face-matching technology
ID: Intellectual disability
IQ: Intellectual quotient
