# Supplementary material for: Computer face-matching technology using two-dimensional photographs accurately matches the facial gestalt of unrelated individuals with the same syndromic form of intellectual disability
Source: BMC Biotechnol. 2017 Dec 19;17:90. doi: 10.1186/s12896-017-0410-1 (PMC5735520; doi:10.1186/s12896-017-0410-1)
Supplement: Supplementary file 1 — Imagus FRT Algorithm Performancev1.1.BL.pdf. Data on Face Recognition Algorithm Performance on Face Recognition Grand Challenge (FRGC) Benchmark. A license agreement to use FRGC database was obtained. (DOCX 139 kb) [file 12896_2017_410_MOESM1_ESM.docx]

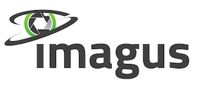


**IMAGUS LEADING THE WAY IN COMPUTER VISION AND BIOMETRIC SOLUTIONS.**

| **Algorithm Performance on FRGC Benchmark**  **imQ Face Recognition** |
| --- |

| **Version 1.1**  This Specification Sheet gives an overview for the measurement of the imQ Face Recognition and Verification performance.  **Revision Date:** September 2017 |  |
| --- | --- |
|  | **Imagus Technology Ptd Ltd**  **ABN** 12 152 800 649  **ADD**  2/A 139 Sandgate Road, Albion QLD 4010 Australia  **PHO** +61 7 3256 0599  **EMA** info@imagus.com.au  **Website:** [www.imagus.com.au](http://www.imagus.com.au) |
|  |  |

# **imQ V2 Face Recognition Performance on NIST FRGC Benchmark**

This benchmark testing is based on the Face Recognition Grand Challenge (FCGC) dataset with 16,028 face images from 4007 subjects.

FRGC was proposed by the **National Institute of Standards and Technology (NIST)** to promote and advance face recognition technology designed to support face recognition efforts in the U.S. Government.

This dataset contains images acquired from both controlled and uncontrolled environments as shown in the following examples.


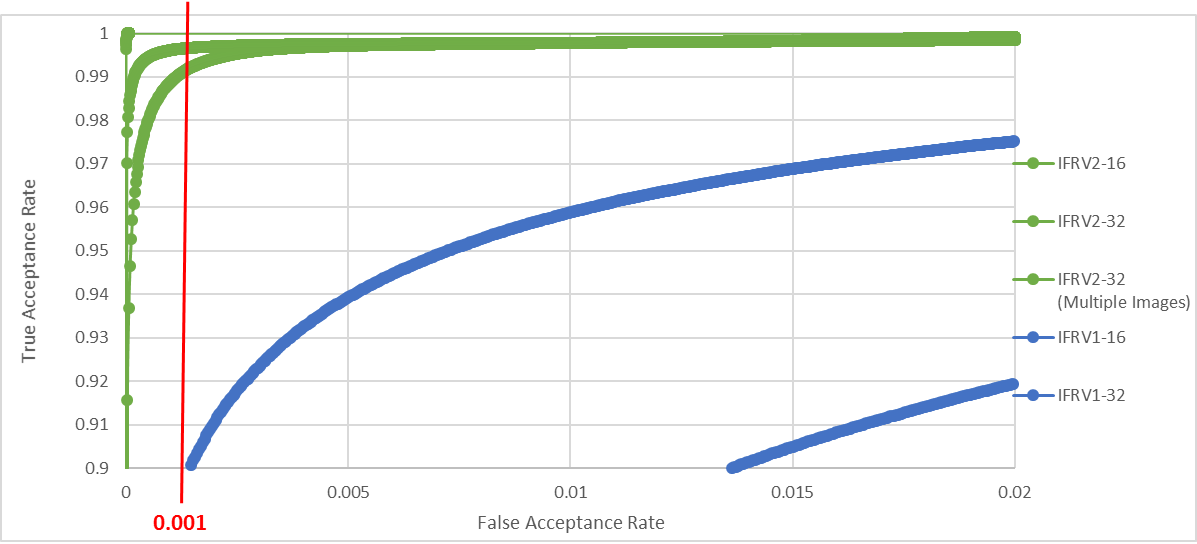


**Legend:**

- IFRV1-16 – Imagus face recognition version 1 – 16 pixels
- IFRV1-32 – Imagus face recognition version 1 – 32 pixels
- IFRV2-16 – Imagus face recognition version 2 – 16 pixels
- IFRV2-32 – Imagus face recognition version 2 – 32 pixels
- IFRV2-32 Multiple Images – Imagus face recognition version 2 – 32 pixels – CCTV or multiple enrolment images (4 images or more).

This graph shows a performance comparison between our current Imagus’ imQ Face Recognition and Verification algorithm IFRV2 (released August 2017) versus our previous algorithm IFRV1. Our new IFRV2 algorithm has been trained on **over 3 million faces** and is based on the latest state-of–the-art **deep learning** techniques. This unique technology has been researched and developed by Imagus for our **face-in-the crowd** surveillance applications. Our new Imagus face recognition and verification technology is vastly more accurate, slightly faster, and offers a huge reduction in memory footprint compared to our earlier award winning technology.

A standard way to compare biometric systems is to measure their False Reject Rate (FRR) at the standard False Acceptance Rate (FAR) of 0.001 or 0.1%. FRR results for each of our algorithms are tabulated below. This table shows that with CCTV or multiple image enrolment our error rate on the FRGC benchmark faces can be considered as negligible (0.01%).

| **imQ Algorithm** | **False Rejection Rate (FRR)  at  False Acceptance Rate  (FAR) = 0.001** |
| --- | --- |
| IFRV1-16 | 0.2415 |
| IFRV1-32 | 0.1111 |
| IFRV2-16 | 0.0111 |
| IFRV2-32 | 0.0039 |
| **IFRV2-32(Multiple Images or CCTV)** | **0.0001** |

- **FAR** is the probability that the system will incorrectly accept an access attempt by an unauthorized user or zero-effort imposter.
- **FRR** is the probability that the system will incorrectly reject an access attempt by an authorized user.

# **imQ V2 Face Recognition Performance Error Rate comparison with NIST FRGC Results**


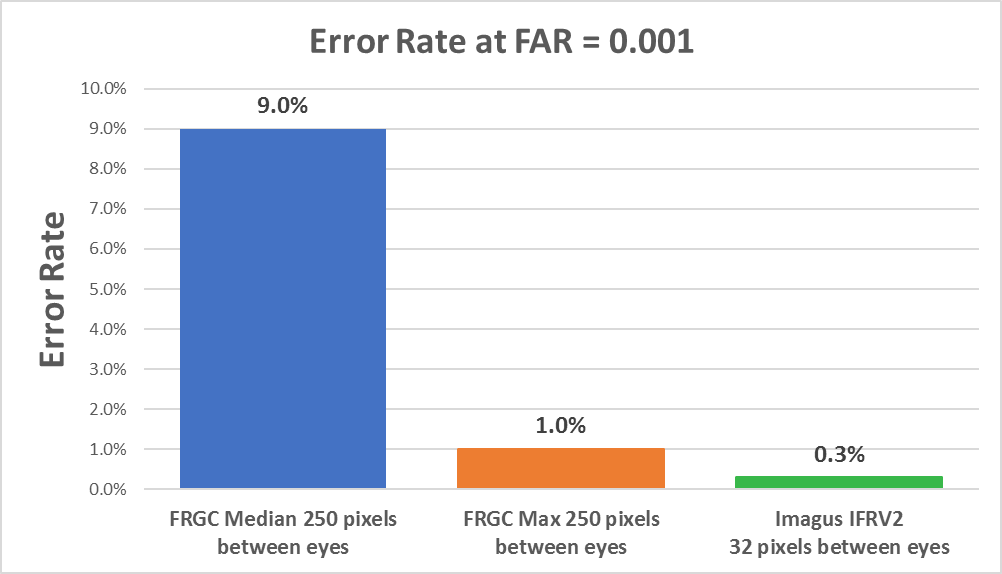


This graph shows the error rate performance comparison between our current Imagus’ imQ Face Recognition and Verification algorithm IFRV2 versus reported NIST FRGC results from other vendors. Note that this graph shows that Imagus now achieves passport photo verification accuracies in a non-cooperative CCTV capture environment using just 32 pixels between the eyes.

# **References**

1. Face Recognition Vendor Tests, <https://www.nist.gov/itl/iad/image-group/face-recognition-vendor-test-frvt-2006>
2. Preliminary Face Recognition Grand Challenge Results, https://www.nist.gov/publications/preliminary-face-recognition-grand-challenge-results
