# Supplementary material for: Computer face-matching technology using two-dimensional photographs accurately matches the facial gestalt of unrelated individuals with the same syndromic form of intellectual disability
Source: BMC Biotechnol. 2017 Dec 19;17:90. doi: 10.1186/s12896-017-0410-1 (PMC5735520; doi:10.1186/s12896-017-0410-1)
Supplement: Supplementary file 2 — Concordance between a software-identified top match and whether all three clinical geneticists would have considered this diagnosis based on the image alone. Table S9. Concordance between a software-identified match within the top five closest matches and whether all three clinical geneticists would have considered this diagnosis based on the image alone. Table S10. Concordance between a software-identified match within the top five closest matches and whether all three clinical geneticists would have considered this diagnosis based on the image alone. (DOCX 17 kb) [file 12896_2017_410_MOESM2_ESM.docx]

**Additional file 2**

**Table S8.** Concordance between a software-identified top match and whether all three clinical geneticists would definitely have considered this diagnosis based on the image alone.

| Syndrome | Frequency | Correctly classified by neither | Correctly classified by both | Correctly classified by software, but not clinicians | Correctly classified by clinicians, but not software | McNemars Chi-square | p-value | Kappa |
| --- | --- | --- | --- | --- | --- | --- | --- | --- |
| Williams | 183 | 51 | 42 | 68 | 22 | 23.51 | <.00001 | 0.07 |
| Rubinstein-Taybi | 155 | 46 | 39 | 53 | 17 | 18.51 | 0.00002 | 0.14 |
| Floating Harbor | 61 | 12 | 24 | 18 | 7 | 4.84 | 0.02781 | 0.18 |
| Coffin Lowry | 154 | 54 | 17 | 71 | 12 | 41.94 | <.00001 | 0.01 |
| Kabuki | 195 | 56 | 48 | 58 | 33 | 6.87 | 0.00877 | 0.08 |
| Smith Magenis | 124 | 42 | 21 | 51 | 10 | 27.56 | <.00001 | 0.09 |
| PACS1 | 39 | 33 | 1 | 5 | 0 | 5.00 | 0.02535 | 0.25 |
| Kleefstra | 128 | 55 | 5 | 67 | 1 | 64.06 | <.00001 | 0.05 |
| Koolan-de Vries | 120 | 37 | 19 | 47 | 17 | 14.06 | 0.00018 | -0.03 |
| Cornelia de Lange | 441 | 76 | 196 | 133 | 36 | 55.67 | <.00001 | 0.21 |

**Table S9.** Concordance between a software-identified match within the top five closest matches and whether all three clinical geneticists would definitely have considered this diagnosis based on the image alone.

| **Syndrome** | Frequency | Correctly classified by neither | Correctly classified by both | Correctly classified by software, but not clinicians | Correctly classified by clinicians, but not software | McNemars Chi-square | p-value | Kappa |
| --- | --- | --- | --- | --- | --- | --- | --- | --- |
| Williams | 183 | 6 | 63 | 113 | 1 | 110.04 | <.00001 | 0.02 |
| Rubinstein-Taybi | 155 | 6 | 56 | 93 | 0 | 93.00 | <.00001 | 0.04 |
| Floating Harbor | 61 | 1 | 29 | 29 | 2 | 23.52 | <.00001 | -0.03 |
| Coffin Lowry | 154 | 21 | 29 | 104 | 0 | 104.00 | <.00001 | 0.07 |
| Kabuki | 195 | 5 | 78 | 109 | 3 | 100.32 | <.00001 | 0.01 |
| Smith Magenis | 124 | 2 | 31 | 91 | 0 | 91.00 | <.00001 | 0.01 |
| PACS1 | 39 | 20 | 1 | 18 | 0 | 18.00 | 0.00002 | 0.05 |
| Kleefstra | 128 | 18 | 4 | 104 | 2 | 98.15 | <.00001 | -0.02 |
| Koolan-de Vries | 120 | 21 | 33 | 63 | 3 | 54.55 | <.00001 | 0.11 |
| Cornelia de Lange | 441 | 17 | 226 | 192 | 6 | 174.73 | <.00001 | 0.06 |

**Table S10.** Concordance between a software-identified match within the top five closest matches and whether all three clinical geneticists would definitely have considered this diagnosis based on the image alone.

| Syndrome | Frequency | Correctly classified by neither | Correctly classified by both | Correctly classified by software, but not clinicians | Correctly classified by clinicians, but not software | McNemars Chi-square | p-value | Kappa |
| --- | --- | --- | --- | --- | --- | --- | --- | --- |
| Williams | 183 | 3 | 63 | 116 | 1 | 113.03 | <.00001 | 0.01 |
| Rubinstein-Taybi | 155 | 0 | 56 | 99 | 0 | . | . | 0.00 |
| Floating Harbor | 61 | 1 | 29 | 29 | 2 | 23.52 | <.00001 | -0.03 |
| Coffin Lowry | 153 | 7 | 29 | 117 | 0 | 117.00 | <.00001 | 0.02 |
| Kabuki | 195 | 2 | 80 | 112 | 1 | 109.04 | <.00001 | 0.00 |
| Smith Magenis | 124 | 1 | 31 | 92 | 0 | 92.00 | <.00001 | 0.01 |
| PACS1 | 39 | 11 | 1 | 27 | 0 | 27.00 | <.00001 | 0.02 |
| Kleefstra | 128 | 9 | 6 | 113 | 0 | 113.00 | <.00001 | 0.01 |
| Koolan-de Vries | 120 | 10 | 35 | 74 | 1 | 71.05 | <.00001 | 0.06 |
| Cornelia de Lange | 441 | 8 | 231 | 201 | 1 | 198.02 | <.00001 | 0.04 |
